# Identification of an immune-related long non-coding RNA signature and nomogram as prognostic target for muscle-invasive bladder cancer

**DOI:** 10.18632/aging.103369

**Published:** 2020-06-24

**Authors:** Yuxuan Song, Donghui Jin, Jingyi Chen, Zhiwen Luo, Guangyuan Chen, Yongjiao Yang, Xiaoqiang Liu

**Affiliations:** 1 Department of Urology, Tianjin Medical University General Hospital, Tianjin 300052, China; 2 Department of Cardiothoracic Surgery, Tianjin Medical University General Hospital, Tianjin 300052, China; 3 Department of Gastroenterology and Institute of Clinical Molecular Biology, Peking University People’s Hospital, Beijing 100044, China; 4 Department of Hepatobiliary Surgery, National Cancer Center, National Clinical Research Center for Cancer, Cancer Hospital, Chinese Academy of Medical Sciences and Peking Union Medical College, Beijing 100021, China; 5 The Second Clinical Medical School, Nanchang University, Nanchang 330006, Jiangxi, China; 6 Department of Urology, The Second Hospital of Tianjin Medical University, Tianjin 300211, China

**Keywords:** muscle-invasive bladder cancer, lncRNA signature, immune-related, prognostic model, nomogram

## Abstract

To identify an immune-related prognostic signature based on long non-coding RNAs (lncRNAs) and find immunotherapeutic targets for bladder urothelial carcinoma, we downloaded RNA-sequencing data from The Cancer Genome Atlas (TCGA) dataset. Functional enrichment analysis demonstrated bladder urothelial carcinoma was related to immune-related functions. We obtained 332 immune-related genes and 262 lncRNAs targeting immune-related genes. We constructed a signature based on eight lncRNAs in training cohort. Patients were classified as high-risk and low-risk according to signature risk score. High-risk patients had poor overall survival compared with low-risk patients (*P* < 0.001). Multivariate Cox regression suggested the signature was an independent prognostic indicator. The findings were further validated in testing, entire TCGA and external validation cohorts. Gene set enrichment analysis indicated significant enrichment of immune-related phenotype in high-risk group. Immunohistochemistry and online analyses validated the functions of 4 key immune-related genes (LIG1, TBX1, CTSG and CXCL12) in bladder urothelial carcinoma. Nomogram proved to be a good classifier for muscle-invasive bladder cancer through combining the signature. In conclusion, our immune-related prognostic signature and nomogram provided prognostic indicators and potential immunotherapeutic targets for muscle-invasive bladder cancer.

## INTRODUCTION

Bladder urothelial carcinoma (BCa) has been identified as the ninth most common malignant neoplasm all over the world [1, 2]. More than 199,000 people died of it and over 549,000 cases were newly diagnosed in 2018 [1, 2]. In the past twenty years, the number of BCa incident cases is growing globally and the BCa burden may ascend in the future due to aging of population and environmental pollution [3, 4]. Although various methods including transurethral resection, radical cystectomy, radiotherapy and chemotherapy are performed in treatment of BCa patients, BCa is aggressive and has a high risk of recurrence, progression, metastasis and poor prognosis [5–7]. The risk of recurrence within 5 years after initial treatment ranges from 50% to 90% in non-muscle-invasive bladder cancer (NMIBC) [2]. The invasion and metastasis of muscle-invasive bladder cancer (MIBC) are vital causes of recurrence and poor prognosis [8, 9]. Hence, it is critical that we should explore and develop reliable prognostic biomarkers to provide prognostic predictors and treatment targets for BCa, which could improve the prognosis of BCa patients.

Recently, lots of evidence shows that disorders of immune system process and immune response play a critical role in tumor microenvironment [10]. Through perturbing the molecular signal and activating the immune response, immune cells could suppress tumor recurrence, progression and metastasis [11, 12]. However, some tumor cells could avoid detection by the immune system, suppress immune response and escape from immune elimination to induce tumor invasion and metastasis [13, 14]. It was reported that dysregulation of immune status induced by tumors might be associated with glioblastoma progression [15, 16]. In the meantime, the immune microenvironment of pancreatic cancer is highly suppressed by immunosuppressive macrophages and myeloid-derived suppressive cells [14]. As to BCa, it is clear from the evidence considered that immune system is highly active in the microenvironment of BCa. Nevertheless, some of these activities are greatly counter-productive and pro-tumorigenic [17].

Long non-coding RNAs (lncRNAs) are a series of RNAs without protein-coding capacity and their lengths are over 200 nucleotides (bp) [18]. Abundant evidence exists to suggest that lncRNAs contribute to tumor development and metastasis through activating immune system process and immune response including antigen release, antigen presentation, immune cell differentiation, immune cells migration, T cells infiltration and recognition and killing of cancer cells [10, 19]. LncRNA CECR7 (cat eye syndrome chromosome region, candidate 7) activates the expression of CTLA4 (cytotoxic T-lymphocyte-associated protein 4) by targeting miR-429 in diabetic pancreatic cancer, which suggested that lncRNAs are involved in immune cell differentiation [20]. Another research revealed that, lnc-sox5 is pivotal in immune cells infiltration in colorectal cancer (CRC). Lnc-sox5 knock-down can directly increase the activity of regulatory T cells and their cytotoxicity is also dramatically enhanced in CRC [21]. In addition, bioinformatics analyses based on The Cancer Genome Atlas (TCGA) database demonstrated that lncRNAs are strongly implicated in the carcinogenesis of BCa through immune-associated pathways [22].

Therefore, aberrantly expressed lncRNAs may be potential prognostic biomarkers for BCa patients and may be served as potential therapeutic targets. As a consequence, based on the gene expression profile of high-throughput sequencing data obtained from TCGA, we carried out this present study in order to explore lncRNAs targeting immune-related genes and further construct an immune-related prognostic signature for MIBC patients.

## RESULTS

**Figure 1:**
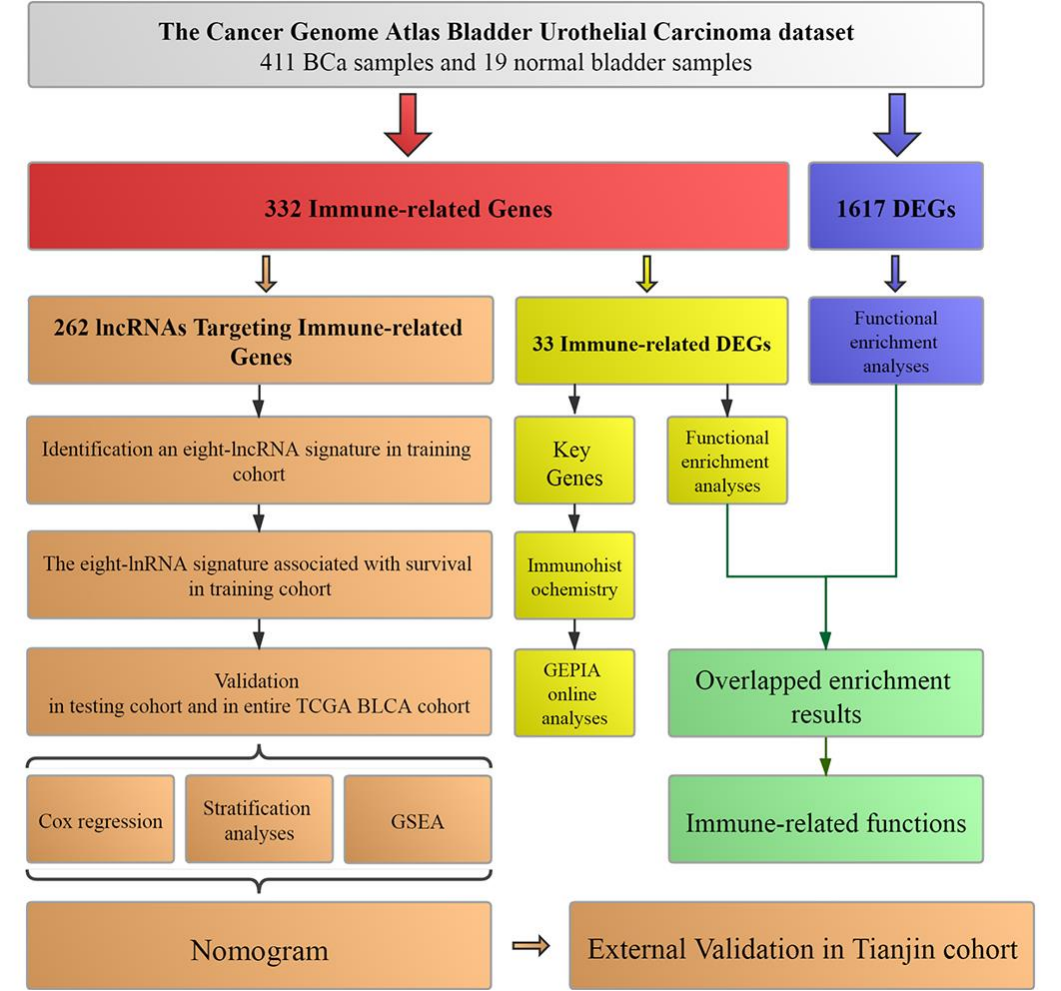
**Workflow of this study.** The study was carried out in TCGA (The Cancer Genome Atlas) BLCA (Bladder Urothelial Carcinoma) dataset. Immune-related genes were extracted from Molecular Signatures Database v4.0. LncRNAs targeting immune-related genes were identified according to Pearson correlation. DEGs (differentially expressed genes) were calculated between BCa (bladder urothelial carcinoma) samples and normal bladder samples in TCGA BLCA dataset. The training cohort was used to identify the lncRNAs targeting immune-related genes and establish a prognostic signature based on the prognostic lncRNAs. The prognosis analysis was validated in the testing cohort, entire TCGA BLCA cohort and Tianjin validation cohort, respectively. Nomogram was constructed by including the immune-related signature and other prognosis-related clinical features in training cohort. Immunohistochemistry from THPA (The Human Protein Atlas) and online analyses from GEPIA (Gene Expression Profiling Interactive Analysis) were used to validate four key immune-related genes (CTSG, CXCL12, LIG1 and TBX1). Functional enrichment analyses were utilized to explore immune-related functions.

The flowchart of the present study was summarized in Figure 1.

### Identification of differentially expressed genes (DEGs) and immune-related DEGs in TCGA BLCA dataset

**Figure 2:**
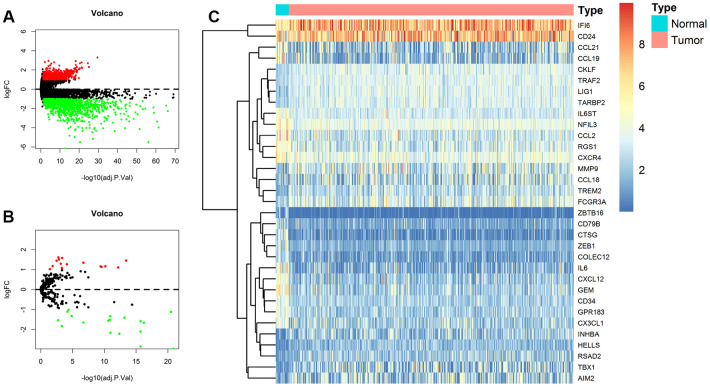
**Identification of differentially expressed genes (DEGs) and immune-related DEGs in TCGA (The Cancer Genome Atlas) BLCA (Bladder Urothelial Carcinoma) dataset.** (**A**) Volcano plot of all DEGs; (**B**) Volcano plot of immune-related DEGs; (**C**) Heat map for immune-related DEGs.

The TCGA BLCA (Bladder Urothelial Carcinoma) dataset was processed with differential expression analysis of all genes. According to the criteria mentioned above, a total of 1617 DEGs including 536 up-regulated and 1081 down-regulated genes between 411 BCa samples and 19 normal bladder samples were selected for further analyses (Figure 2A).

In addition, 332 immune-related genes were identified from Molecular Signatures Database v4.0 and were also analyzed with differential expression analysis. A total of 33 immune-related DEGs were screened out, among which 18 were up-regulated and 15 were down-regulated as shown in the volcano plot and heat map (Figure 2B, 2C).

### Functional enrichment analysis

**Figure 3:**
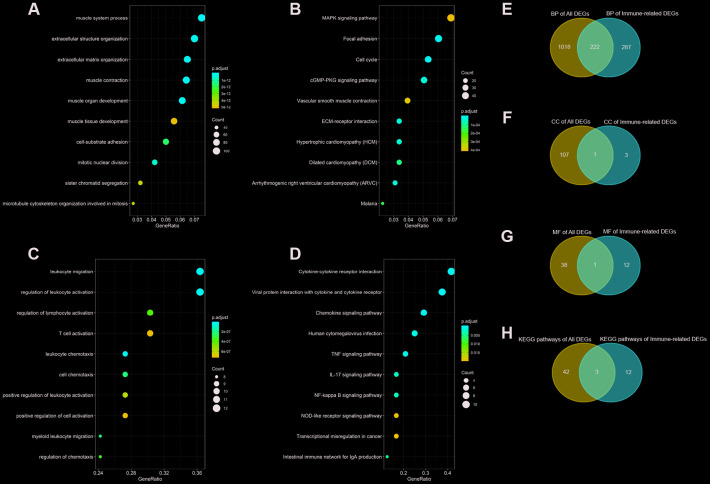
**GO (Gene Ontology) functional and KEGG (Kyoto Encyclopedia of Genes and Genomes) pathway enrichment analyses of differentially expressed genes (DEGs) and immune-related DEGs.** (**A**) Top 10 BP (biological process) terms of all DEGs; (**B**) Top 10 KEGG pathways of all DEGs; (**C**) Top 10 BP terms of immune-related DEGs; (**D**) Top 10 KEGG pathways of immune-related DEGs; (**E**) Venn diagram for overlapped BP terms; (**F**) Venn diagram for overlapped CC (cell component) terms; (**G**) Venn diagram for overlapped MF (molecular function) terms; (**H**) Venn diagram for overlapped MF (molecular function) KEGG pathways.

We performed Gene Ontology (GO) and Kyoto Encyclopedia of Genes and Genomes (KEGG) pathway enrichment analyses to determine the biological function and pathways involved in the 1617 DEGs in TCGA BLCA dataset. The top 10 enrichment items of biological process (BP) and KEGG pathway analyses were illustrated in Figure 3A, 3B, separately.

At the same time, we also analyzed the 33 immune-related DEGs with GO and KEGG pathway enrichment analyses (Figure 3C, 3D).

Next, to validate and confirm that BCa is closely immune-related, we adopted Venn diagram to obtain overlapped enrichment results between all DEGs enrichment analysis and immune-related DEGs enrichment analysis. We found that they both had enrichments in negative regulation of immune system process (GO:0002683), G protein-coupled receptor binding (GO:0001664) and transcriptional misregulation in cancer (hsa05202), which indicated that the development of BCa was associated with immune system and immune response (Figure 3E).

### Clinical characteristics of TCGA BLCA patients

A total of 389 BCa patients were involved in construction and validation of the lncRNA signature. They were randomly assigned to a training cohort (n = 195) for construction and a testing cohort (n = 194) for validation. Clinical characteristics of 389 BCa patients were displayed in Table 1.

**Table 1:** Clinical characteristics of 389 bladder urothelial carcinoma patients involved in identification and validation of the 8-lncRNA prognostic signature.

**Characteristics**	**Entire TCGA BLCA cohort (N=389)**	**Detailed data**		***P*-value**
		**Training cohort (N=195)**	**Testing cohort (N=194)**	
Age at diagnosis (years)				
<65	147 (37.8%)	64 (32.8%)	83 (42.8%)	0.055
≥65	242 (62.2%)	131 (67.2%)	111 (57.2%)	
Gender				
Male	287 (73.8%)	140 (71.8%)	147 (75.8%)	0.372
Female	102 (26.2%)	55 (28.2%)	47 (24.2%)	
Histological grade				
Low grade	25 (6.4%)	8 (4.1%)	17 (8.8%)	0.061
High grade	364 (93.6%)	187 (95.9%)	177 (91.2%)	
TNM (UICC) stage				
Stage I	2 (0.5%)	2 (1.0%)	0 (0.0%)	0.42
Stage II	128 (32.9%)	68 (34.9%)	60 (30.9%)	
Stage III	136 (35.0%)	66 (33.8%)	70 (36.1%)	
Stage IV	123 (31.6%)	59 (30.3%)	64 (33.0%)	

^**a**^ Chi-square test.

### Identification of the 8-lncRNA prognostic signature in training cohort

Pearson correlation analysis identified that 262 lncRNAs were correlated with the 18 up-regulated immune-related DEGs or 15 down-regulated immune-related DEGs in the TCGA BLCA dataset (|R| > 0.5 and *P* < 0.01). And the 262 lncRNAs were lncRNAs targeting immune-related genes. Next, we used univariate Cox regression analysis method to identify prognosis-related lncRNAs from the 262 lncRNAs in the training cohort and univariate Cox regression analysis identified 21 prognosis-related lncRNAs (*P* < 0.01). Then, we used stepwise selection with Akaike information criteria (AIC) from the 21 prognosis-related lncRNAs to select the optimal model. Ultimately, we selected 8 lncRNAs with the smallest AIC value to construct the predictive model002E

Details of the 8 lncRNAs with their gene symbols, Ensembel IDs, descriptions, coefficients and results of univariate Cox regression analysis were summarized in Table 2. Among these eight lncRNAs, two are deleterious lncRNAs with univariate Cox hazard ratio (HR) > 1 (WNT5A-AS1 and AL136084.3), which indicated that patients with high expression of the two lncRNAs might have a poor survival time. The other six are protective lncRNAs with univariate Cox HR < 1 (MIF-AS1, AC008735.2, AL357033.4, LINC00649, AC099343.2 and USP30-AS1), which indicated that high expression of the six lncRNAs might result in a better survival time.

**Table 2:** The eight lncRNAs identified from Cox regression analysis.

**Gene symbol**	**Ensembel ID**	**Description**	**Coefficient**	**Univariate Cox regression analysis**		
				**HR**	**95%CI**	***P***-**value**
WNT5A-AS1	ENSG00000244586	WNT5A Antisense RNA 1	0.231	1.335	1.113-1.601	0.002
AL136084.3	ENSG00000270412	-	0.420	1.686	1.194-2.380	0.003
MIF-AS1	ENSG00000218537	MIF Antisense RNA 1	-0.564	0.460	0.258-0.820	0.009
AC008735.2	ENSG00000267523	-	-0.388	0.672	0.510-0.885	0.005
AL357033.4	ENSG00000277496	-	-0.456	0.576	0.403-0.823	0.002
LINC00649	ENSG00000237945	Long Intergenic Non-Protein Coding RNA 649	-0.661	0.486	0.290-0.817	0.006
AC099343.2	ENSG00000270426	-	-0.633	0.280	0.143-0.548	<0.001
USP30-AS1	ENSG00000256262	USP30 Antisense RNA 1	-0.263	0.697	0.536-0.905	0.007

Based on the expression of these eight lncRNAs for overall survival (OS) prediction, we established a risk score of the 8-lncRNA signature with the following formula:

Risk score = (0.231 × Expression_WNT5A-AS1_) + (0.420 × Expression_AL136084.3_) + (-0.564 × Expression_MIF-AS1_) + (-0.388 × Expression_AC008735.2_) + (-0.456 × Expression_AL357033.4_) + (-0.661 × Expression_LINC00649_) + (-0.633 × Expression_AC099343.2_) + (-0.263 × Expression_USP30-AS1_).

### Evaluating the predictive power of the 8-lncRNA prognostic signature in training cohort

**Figure 4:**
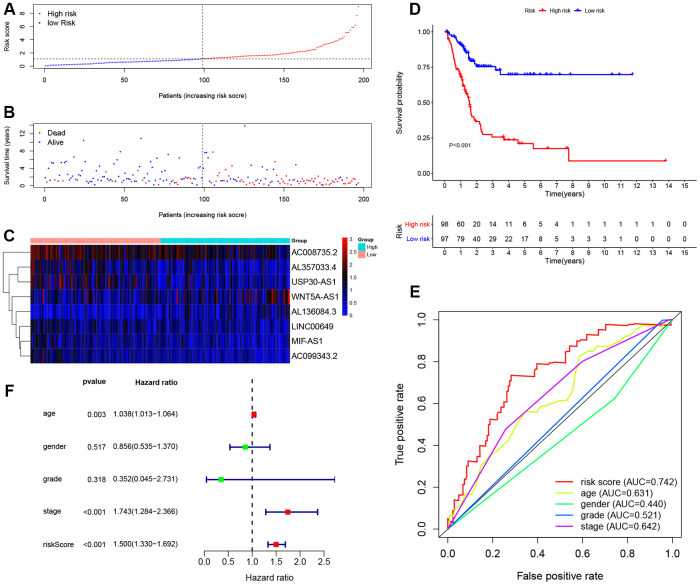
**Evaluating the predictive power of the 8-lncRNA immune-related signature in the training cohort.** (**A**–**C**) Distribution of risk score, survival status, and lncRNA expression of patients in the training cohort; (**D**) Kaplan-Meier survival curve of the high-risk and low-risk groups in the training cohort; (**E**) Time-dependent ROC (receiver operating characteristic) curves and AUC (area under curve) based on the training cohort for 5-year overall survival; (**F**) Forest plot for multivariate Cox regression analysis.

195 BCa patients in training cohort were divided into high-risk (n = 98) and low-risk (n = 97) groups determined by the median value of 8-lncRNA signature risk score. The risk score, survival time and survival status of each patient in training cohort were illustrated in Figure 4A, 4B. The heat map of the expression of the eight lncRNAs was displayed in Figure 4C.

The Kaplan-Meier (KM) survival curve of low-risk and high-risk groups in training cohort was shown in Figure 4D. The survival time of the low-risk group was significantly higher than that of the high-risk group (*P* < 0.001). In addition, time-dependent receiver operating characteristic (ROC) curves of 8-lncRNA prognostic signature and other clinical characteristics were plotted. The area under curve (AUC) of 8-lncRNA prognostic signature risk score was 0.742, which was better than other clinical characteristics in Figure 4E.

Furthermore, multivariate Cox regression analyses of age, gender, Union for International Cancer Control (UICC) stage, histological grade and 8-lncRNA prognostic signature were evaluated in Figure 4F. Risk score of the 8-lncRNA signature (HR = 1.500, 95%CI = 1.330-1.692, *P* < 0.001) might be considered as an independent prognostic indicator of OS (Table 3).

**Table 3:** Univariate and multivariate Cox regression analyses of the training, testing, and entire TCGA BLCA cohorts.

**Variables**	**Univariate analysis**			**Multivariate analysis**		
	**HR**	**95%CI**	***P*-value**	**HR**	**95%CI**	***P*-value**
**Training cohort**						
8-lncRNA Risk score (continuous variables)	**1.514**	**1.356-1.691**	**<0.001**	**1.500**	**1.330-1.692**	**<0.001**
Age at diagnosis (≥65 years vs. <65 years)	**1.042**	**1.018-1.067**	**0.001**	**1.038**	**1.013-1.064**	**0.003**
Gender (Male vs. Female)	0.761	0.480-1.207	0.246	0.856	0.535-1.370	0.517
Histological grade (High grade vs. Low grade)	1.708	0.235-12.389	0.597	0.352	0.045-2.731	0.318
TNM (UICC) stage (Stage IV+Stage III vs. Stage II+Stage I)	**1.865**	**1.412-2.464**	**<0.001**	**1.743**	**1.284-2.366**	**<0.001**
**Testing cohort**						
8-lncRNA Risk score (continuous variables)	**1.141**	**1.100-1.184**	**<0.001**	**1.137**	**1.090-1.186**	**<0.001**
Age at diagnosis (≥65 years vs. <65 years)	**1.029**	**1.003-1.055**	**0.027**	1.015	0.989-1.042	0.250
Gender (Male vs. Female)	1.011	0.584-1.750	0.968	0.814	0.463-1.432	0.476
Histological grade (High grade vs. Low grade)	1.340	0.322-5.578	0.687	0.588	0.130-2.665	0.491
TNM (UICC) stage (Stage IV+Stage III vs. Stage II+Stage I)	**1.623**	**1.185-2.223**	**0.003**	**1.749**	**1.238-2.471**	**0.002**
**Entire TCGA BLCA cohort**						
8-lncRNA Risk score (continuous variables)	**1.042**	**1.037-1.047**	**<0.001**	**1.047**	**1.041-1.053**	**<0.001**
Age at diagnosis (≥65 years vs. <65 years)	**1.037**	**1.020-1.055**	**<0.001**	**1.043**	**1.024-1.062**	**<0.001**
Gender (Male vs. Female)	0.859	0.604-1.220	0.395	**0.636**	**0.441-0.915**	**0.015**
Histological grade (High grade vs. Low grade)	2.090	0.977-4.470	0.057	1.026	0.441-2.385	0.952
TNM (UICC) stage (Stage IV+Stage III vs. Stage II+Stage I)	**1.848**	**1.496-2.282**	**<0.001**	**1.935**	**1.500-2.496**	**<0.001**

HR hazard ratio, CI confidence interval.

### Validation of the 8-lncRNA prognostic signature in testing cohort and entire TCGA BLCA cohort

**Figure 5:**
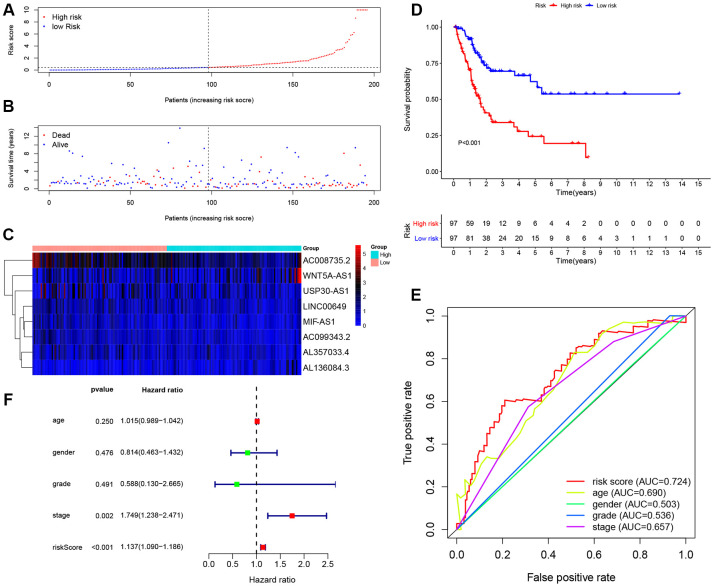
**Evaluating the predictive power of the 8-lncRNA immune-related signature in the testing cohort.** (**A**–**C**) Distribution of risk score, survival status, and lncRNA expression of patients in the testing cohort; (**D**) Kaplan-Meier survival curve of the high-risk and low-risk groups in the testing cohort; (**E**) Time-dependent ROC (receiver operating characteristic) curves and AUC (area under curve) based on the testing cohort for 5-year overall survival; (**F**) Forest plot for multivariate Cox regression analysis.

**Figure 6:**
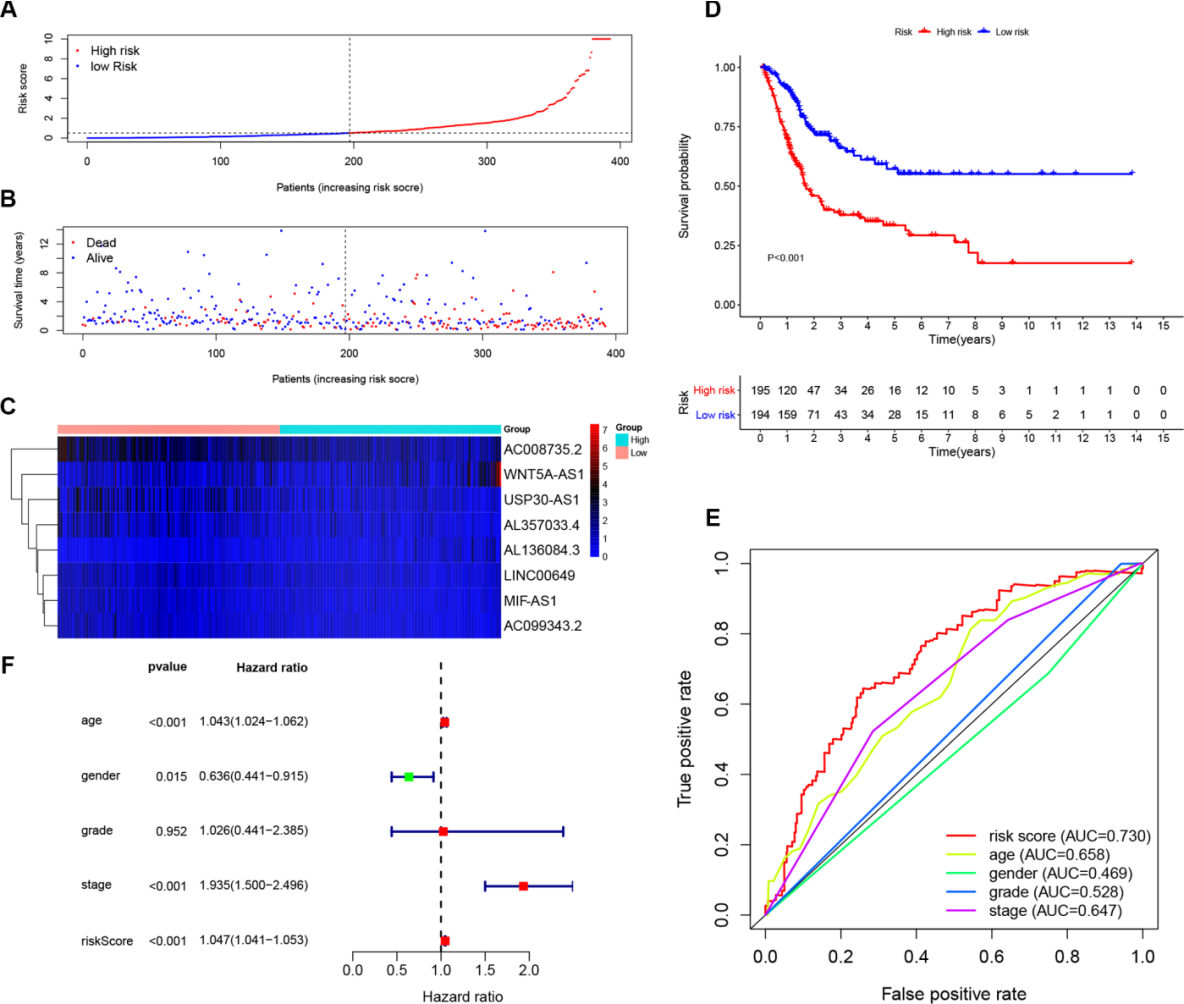
**Evaluating the predictive power of the 8-lncRNA immune-related signature in the entire TCGA (The Cancer Genome Atlas) BLCA (Bladder Urothelial Carcinoma) cohort.** (**A**–**C**) Distribution of risk score, survival status, and lncRNA expression of patients in the entire TCGA BLCA cohort; (**D**) Kaplan-Meier survival curve of the high-risk and low-risk groups in the entire TCGA BLCA cohort; (**E**) Time-dependent ROC (receiver operating characteristic) curves and AUC (area under curve) based on the entire TCGA BLCA cohort for 5-year overall survival; (**F**) Forest plot for multivariate Cox regression analysis.

In order to further confirm the predictive power and stability of the 8-lncRNA signature in predicting the OS of BCa patients, we validated it in testing cohort (n = 194) and entire TCGA BLCA cohort (n = 389) (Figures 5 and 6). Risk scores of the 8-lncRNA signature were also calculated with the above mentioned formula.

The KM survival curve of low-risk and high-risk groups in testing cohort was shown in Figure 5D. The survival time of the low-risk group was significantly higher than that of the high-risk group (*P* < 0.001). In addition, time-dependent ROC curves of 8-lncRNA prognostic signature and other clinical characteristics were plotted. Consistent with the finding in training cohort, the AUC of 8-lncRNA prognostic signature risk score was 0.724, which was better than other clinical characteristics in Figure 5E. Multivariate Cox regression analysis revealed that risk score of the 8-lncRNA signature (HR = 1.137, 95%CI = 1.090-1.186, *P* < 0.001) might be an independent prognostic indicator in Figure 5F and Table 3. Similar results of validation were obtained in entire TCGA BLCA cohort.

### Survival and Clinical characteristics with 8-lncRNA prognostic signature in entire TCGA BLCA cohort

**Figure 7:**
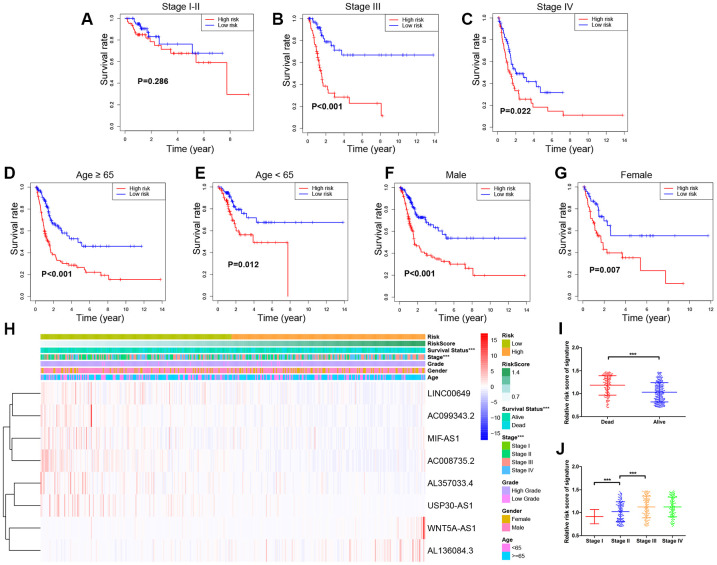
**Stratified survival analyses and Clinical characteristics with 8-lncRNA prognostic signature in the entire TCGA (The Cancer Genome Atlas) BLCA (Bladder Urothelial Carcinoma) cohort.** (**A**–**G**) Kaplan-Meier survival curves in subgroups stratified by different clinical characteristics; (**H**) Distribution of clinicopathologic features, and lncRNA expression in low-risk and high-risk groups; (**I**) Risk score comparison between alive and dead patients; (**J**) Risk score comparison between different tumor stages. *** *P*-value < 0.005.

To further validate the prognostic value and explore the wide applicability of the immune-related signature, we performed survival analyses through stratification analysis based on entire TCGA BLCA cohort (Figure 7A–7G). Samples in the high-risk group had poor survival compared with those in the low-risk group among stage III (*P* < 0.001) and stage IV (*P* = 0.022) patients. Similar significant results were revealed in different age groups and different gender groups.

Clinical characteristics exhibited distributed patterns corresponding to the risk score of 8-lncRNA prognostic signature on the entire TCGA BLCA cohort (Figure 7H). We found that dead samples more likely gathered in the high-risk group, indicating that samples with high risk score might have worse survival (*P* < 0.005) (Figure 7I). In addition, samples with higher UICC stages (stage III and stage IV) were more likely to have higher risk scores than those with lower stages (stage I and stage II) (*P* < 0.005) (Figure 7J), which confirmed that higher scores of the 8-lncRNA prognostic signature might be significantly associated with the progression of BCa.

### Principal component analysis (PCA) and Gene set enrichment analysis (GSEA)

**Figure 8:**
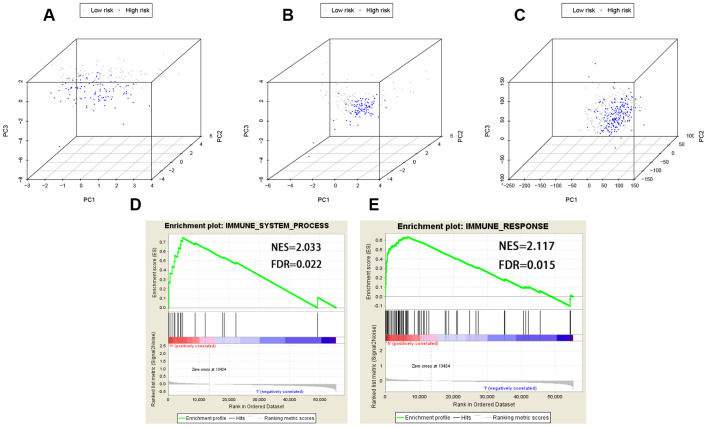
**PCA (Principal components analysis) and GSEA (Gene set enrichment analysis).** PCA based on the eight lncRNAs indicated low-risk and high-risk groups were generally distributed in two different directions in (**A**) the training cohort and (**B**) the testing cohort, respectively; (**C**) PCA based on the whole gene set indicated these two groups did not show significant distinctions; (**D**, **E**) GSEA indicated significant enrichment of immune-related phenotype in the high-risk group patients. FDR false discovery rate; NES normalized enrichment score.

PCA was performed to explore the different distribution patterns between low-risk and high-risk groups according to the eight lncRNAs targeting immune-related genes and the whole gene set. Based on the eight lncRNAs, low-risk and high-risk groups were significantly distributed in two different directions. That is to say, the eight lncRNAs were used to separate BCa patients into two sections, indicating that the BCa patients in the low-risk group was quite distinguished from those in the high-risk group (Figure 8A, 8B). However, these two groups did not show significant distinctions when PCA was performed based on the whole gene set (Figure 8C).

GSEA was further utilized to validate the functional annotation between high-risk and low-risk groups in entire TCGA cohort. The DEGs between the two groups were enriched in immune system process pathway (FDR [False discovery rate] = 0.022, NES [normalized enrichment score] = 2.033) and immune response pathway (FDR = 0.015, NES = 2.117) (Figure 8D, 8E). In summary, the immune-related prognostic signature based on the eight lncRNAs targeting immune-related genes was significantly associated with the immune status of BCa, and high risk score of the signature was more likely to activate immune-related pathways in BCa patients.

### Validation based on immunohistochemistry and survival analysis of immune-related DEGs

**Figure 9:**
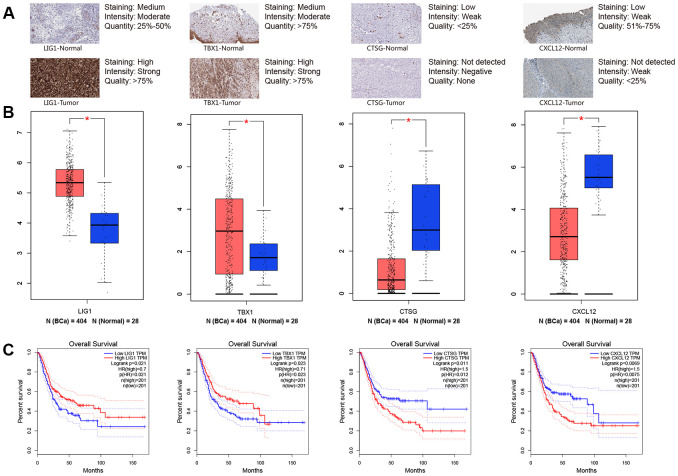
**Immunohistochemistry from THPA (The Human Protein Atlas) and online analyses from GEPIA (Gene Expression Profiling Interactive Analysis) were used to explore four key immune-related genes (LIG1, TBX1, CTSG and CXCL12).** (**A**) Immunohistochemistry between normal bladder tissues and BCa (bladder urothelial carcinoma) tissues; (**B**) Gene expression level between normal bladder tissues and BCa tissues for LIG1, TBX1, CTSG and CXCL12, respectively; (**C**) Kaplan-Meier survival curve of LIG1, TBX1, CTSG and CXCL12, respectively. * *P*-value < 0.05.

In order to further validate the immune-related prognostic signature, we compare the expression of 4 immune-related DEGs including CTSG, CXCL12, LIG1 and TBX1 between normal bladder tissues and BCa tissues from The Human Protein Atlas (THPA). Immunohistochemistry indicated that the expression levels of CTSG and CXCL12 are down-regulated in BCa tissues, while LIG1 and TBX1 are up-regulated in BCa tissues. These results confirmed that there are differences in the expression of the four immune-related DEGs between BCa tissues and normal bladder tissues (Figure 9A).

We continued to extract data from Gene Expression Profiling Interactive Analysis (GEPIA) database and KM curves of the four immune-related DEGs revealed that higher expression levels of CTSG (*P* = 0.011) and CXCL12 (*P* = 0.0069) are associated with worse OS time of BCa. However, higher expression levels of LIG1 (*P* = 0.021) and TBX1 (*P* = 0.023) contributed to a better prognosis compared with lower expression levels (Figure 9B).

### Building and validating a predictive nomogram

**Figure 10:**
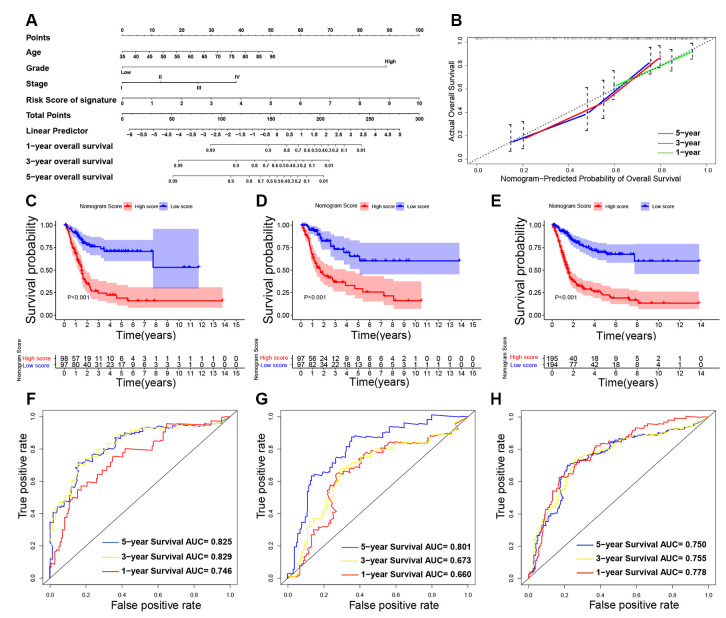
**Building and validating the nomogram to predict prognosis in TCGA (The Cancer Genome Atlas) BLCA (Bladder Urothelial Carcinoma) dataset.** (**A**) The nomogram was constructed based on age, UICC (Union for International Cancer Control) stage, histological grade and the immune-related signature in the training cohort; (**B**) The calibration plot for internal validation of the nomogram; (**C**–**E**) Kaplan-Meier survival curves between high-nomogram-score and low-nomogram-score groups in the training cohort, testing cohort and entire TCGA BLCA cohort, respectively; (**F**–**H**) Time-dependent ROC (receiver operating characteristic) curves and AUC (area under curve) for 1-year, 3-year, and 5-year overall survival based on the training cohort, testing cohort and entire TCGA BLCA cohort, respectively.

Nomogram was constructed by combining the details of age, UICC stage, histological grade, and the 8-lncRNA prognostic signature in training cohort (Figure 10A). By using bootstrap method, calibration plots showed no significant deviation from the ideal for 1-year, 3-year and 5-year survival (Figure 10B). The training cohort (N = 195) was divided into two high-nomogram-score (N = 98) and low-nomogram-score (N = 97) groups by the median value of nomogram score. The KM curve showed that BCa patients in high-nomogram-score group had worse prognosis compared with low-nomogram-score group (*P* < 0.001) (Figure 10C). The AUCs for 1-year, 3-year and 5-year of their time-dependent ROC curves were 0.746, 0.829, and 0.825, respectively (Figure 10F). Combining the immune-related prognostic signature with other prognosis-related clinical factors increased the AUC for predicting OS of BCa patients.

In addition, we also validated the nomogram in testing cohort (n = 194) and in entire TCGA BLCA cohort (n = 389). KM curves and their ROC curves were displayed in Figure 10D, 10E, 10G, 10H.

Table 4 showed the concordance index (c-index) of the immune-related signature and nomogram. Table 5 showed the AUC in time-dependent ROC curves. Both the c-index and AUC are indicators for evaluating the predictive value of prognostic model. The c-index of nomogram exceeded the c-index of immune-related signature in training cohort, testing cohort and entire TCGA BLCA cohort (*P* < 0.05), which indicated the predictive value of nomogram was better than the immune-related signature. In addition, the AUC of nomogram exceeded the AUC of immune-related signature, especially in predicting 5-year OS (*P* < 0.05), which indicated the nomogram had better predictive power in long-term follow-up. Therefore, the nomogram combined the immune-related prognostic signature with other prognosis-related clinical factors and increased the predictive power of OS, which might help to improve clinical management of BCa patients.

**Table 4:** Concordance index (C-index).

	**C-index (95%CI)**		**Z-test**	***P*-value**
	**8-lncRNA Risk score**	**Nomogram**		
**Entire TCGA BLCA cohort**	0.678 (0.626-0.715)	0.727 (0.697-0.748)	**2.303**	**0.021**
**Training cohort**	0.694 (0.659-0.728)	0.734 (0.691-0.756)	**1.975**	**0.048**
**Testing cohort**	0.642 (0.598-0.671)	0.716 (0.662-0.742)	**3.800**	**<0.001**

### External validation of immune-related signature and nomogram in Tianjin cohort

To further confirm the 8-lncRNA signature and nomogram for MIBC, we recruited MIBC patients (n = 72) from Tianjin cohort for validation. Clinical characteristics of enrolled BC patients and controls in Tianjin validation cohort are displayed in Table 6. Quantitative real-time polymerase chain reaction (qRT-PCR) was performed to measure the expression levels of the eight lncRNAs. Risk score was calculated with the previous following formula of signature.

**Table 6:** Characteristics of muscle-invasive bladder cancer (MIBC) patients in Tianjin validation cohort.

**Characteristics**	**MIBC patients (n = 72)**
Age at diagnosis (years)	
<65	25 (34.72%)
≥65	47 (65.28%)
Gender	
Male	53 (73.61%)
Female	19 (26.39%)
Histological grade	
Low grade	10 (13.89%)
High grade	62 (86.11%)
TNM (UICC) stage	
Stage II	19 (26.39%)
Stage III	36 (50%)
Stage IV	17 (23.61%)

**Figure 11:**
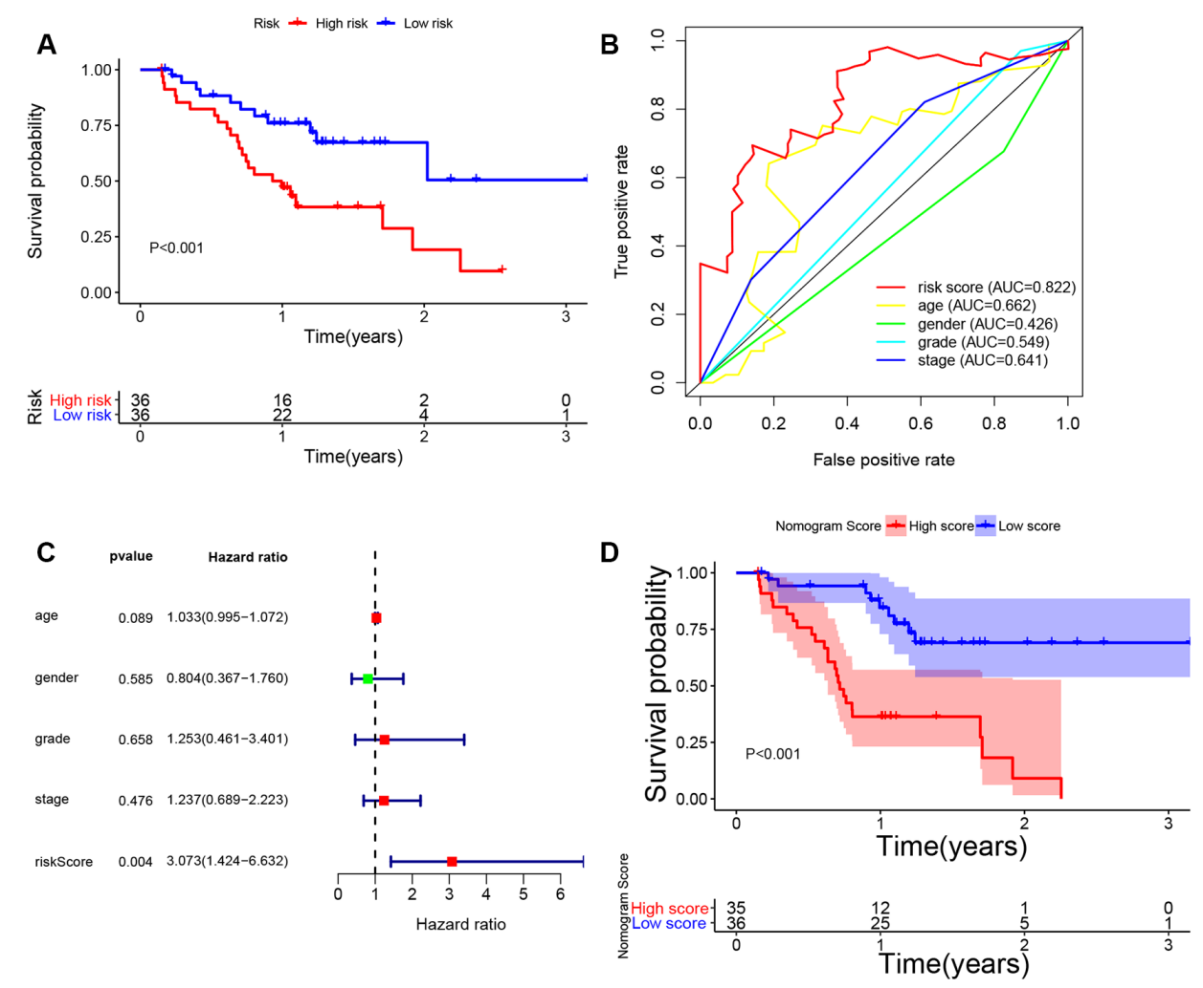
**External validation of 8-lncRNA immune-related signature and nomogram in Tianjin cohort.** (**A**) Kaplan-Meier survival curve of 8-lncRNA immune-related signature in Tianjin validation cohort; (**B**) Time-dependent ROC (receiver operating characteristic) curves and AUC (area under curve) of 8-lncRNA immune-related signature based on Tianjin validation cohort for 3-year overall survival; (**C**) Forest plot for multivariate Cox regression analysis; (D) Kaplan-Meier survival curve of nomogram in Tianjin validation cohort.

The KM survival curve of Tianjin cohort indicated the survival time of the low-risk group was significantly higher than that of the high-risk group in Figure 11A (*P* < 0.001). Consistent with the finding in previous cohorts, time-dependent ROC curves indicated that the AUC of 8-lncRNA prognostic signature risk score was 0.822 (95%CI = 0.793-0.851), which was better than other clinical characteristics in Figure 11B. C-index was 0.723 (95%CI = 0.688-0.751). Furthermore, multivariate Cox regression analysis revealed that risk score of the 8-lncRNA signature (HR = 3.073, 95%CI = 1.424-6.632, *P* = 0.004) might be an independent prognostic indicator in Figure 11C.

Furthermore, we also validated the nomogram in Tianjin cohort (Figure 11D). The KM curve showed that MIBC patients in high-nomogram-score group had worse prognosis compared with low-nomogram-score group (*P* < 0.001). The AUC of nomogram was 0.856 (95%CI = 0.835-0.877) and c-index was 0.798 (95%CI = 0.763-0.817), which increased the predictive power of OS compared with the 8-lncRNA signature (*P* < 0.05). The results in Tianjin validation cohort confirmed the predictive power of immune-related signature and nomogram, which was consistent with the results of TCGA dataset.

## DISCUSSION

Recently, the roles of lncRNAs in the development and prognosis of various tumors have been well investigated in many studies. Aberrantly expressed lncRNAs in cancer can be utilized as biomarkers for diagnosis, prognosis and target therapy [22]. Shen et al. performed a study based on TCGA BLCA dataset identified a panel of 20 key lncRNAs that were most implicated in MIBC prognosis after differential expression analysis and prognostic correlations [22]. Among the 20 lncRNAs, lnc-BOD1-1:7, -1:8, and -1:9, as well as lnc-GCH1-2:1, -2:2, and -2:3 were associated with both immune-related pathways and cancer-associated pathways. High expression level of lnc-CGRRF1-3:1 was mainly correlated with increased immune activity and decreased apoptosis in MIBC. In addition, Cao et al. carried out another study based on TCGA BLCA dataset to explore the tumor immune microenvironment of BCa [23]. They found the abundance ratio of four immune cells including T cell CD4 memory activated, T cell CD4 memory resting, T cell CD8 and natural killer (NK) cell resting was related to BCa survival. T cell CD4 memory resting, T cell CD8, NK cell resting decreased as the increase of UICC stage and lymph node metastasis, which indicated that the four cells are vital in immune infiltration as well as BCa immunotherapy.

As immunotherapy has attracted increasing attention in the field of cancer treatment, the gene expression profile of high-throughput sequencing data have been used in order to explore valuable immune-related biomarkers, identify potential prognostic targets and analyze underlying mechanisms [24, 25]. In the present study, we adopted TCGA BLCA dataset to explore lncRNAs targeting immune-related genes and construct an immune-related signature, which may serve as promising prognostic indicators for BCa and can be applied as promising immune therapeutic targets.

Previous studies have identified that the immune system is significantly associated with the tumor microenvironment, especially tumor immune escape [23]. Immune components in the tumor microenvironment have essential effects on gene expression by tumor tissues and the clinical outcomes [26, 27]. In this study, a total of 332 immune-related genes were identified from TCGA BLCA dataset. According to differential expression analysis, we found 1617 DEGs and 33 immune-related DEGs between BCa samples and normal bladder samples. Negative regulation of immune system process (GO:0002683) is the overlapped GO item based on the results of enrichment analyses of both DEGs and immune-related DEGs, which indicated that the development of BCa was associated with immune system and immune response (Figure 3E).

Next, we established an immune-related prognostic signature based on eight lncRNAs targeting immune-related genes (WNT5A-AS1, AL136084.3, MIF-AS1, AC008735.2, AL357033.4, LINC00649, AC099343.2 and USP30-AS1) in the training cohort. BCa patients were divided into low-risk and high-risk groups according to the median risk score of 8-lncRNA signature and we further found the immune-related signature was able to distinguish samples with a better prognosis or a worse prognosis. Low-risk group had better OS than high-risk group (Figures 4D, 5D and 6D). Results of multivariate Cox regression analyses revealed that higher risk score was an independent poor prognostic indicator of OS (HR > 1) (Table 3, Figures 4F, 5F and 6F). ROC curves showed that the AUCs of the immune-related signature were higher than those of UICC stage and histological grade, indicating that the 8-lncRNA signature may be superior to other clinicopathologic features and serve as better prognostic biomarkers (Figures 4E, 5E and 6E). For external validation of the immune-related signature for MIBC, we recruited 72 MIBC patients and validated these results in Tianjin validation cohort. The KM survival curve, time-dependent ROC curves and multivariate Cox regression analysis were consistent with the results of TCGA dataset, which confirmed the predictive power of the prognostic signature (Figure 11).

In order to explore the wide applicability of the 8-lncRNA signature, we performed survival analyses in different subgroups stratified by UICC stage, age and gender. We observed that the 8-lncRNA signature was able to assess the risk score in various subgroups by accurately dividing the patients into high-risk group with poor survival and low-risk group with good survival (Figure 7B–7G), except for patients with stage I and stage II BCa (Figure 7A). One explanation for this phenomenon is that most of the patients with low stage BCa had favourable prognosis with good survival and the heterogeneity of prognosis among them is small [4]. In addition, we also compared the risk scores between different subgroups and we identified that patients with advanced BCa (stage IV and stage III) had a higher risk score than those low stage BCa (stage II and stage I) (Figure 7J). The above results indicated that the 8-lncRNA prognostic signature could help to distinguish prognosis of patients with different variables and may also predict the tumor progression.

To further validate the 8-lncRNA prognostic signature was tightly associated with immune system, GSEA was carried out between high-risk and low-risk groups. We identified that up-regulation of the immune-related signature was enriched in immune system process pathway (FDR = 0.022, Figure 8D) and immune response pathway (FDR = 0.015, Figure 8E). We also selected four immune-related DEGs including CTSG, CXCL12, LIG1 and TBX1 to explore their functions in BCa tumorigenesis and prognosis. CTSG is reported to activate neutrophil effector functions through release of formyl peptide receptor agonists in inflammatory and immune responses [28, 29]. The CXCL12-CXCR4/CXCR7 chemokine axis activates immune cell migration and inhibits immune resistance in gastrointestinal malignancies [30]. Biallelic et al. found mutations in LIG1 are identified to underlie a spectrum of immune deficiencies by in vitro studies and TBX1 mutation is responsible for most of the congenital immune defect seen in the mouse models and in patients [31, 32]. Immunohistochemistry from THPA and online analyses from GEPIA demonstrated that all the 4 immune-related DEGs differentially expressed between BCa and normal bladder tissues and they were associated with OS of BCa patients (*P* < 0.05, Figure 9).

In addition, our predictive nomogram combining our immune-related signature suggested that AUCs of 1-year, 3-year and 5-year survival in the entire TCGA BLCA cohort are all greater than 0.700, indicating our nomogram and immune-related signature have wide applicability for both long-term and short-term follow-up patients (Figure 10F–10H). In addition, we used c-index and AUC to compare the predictive value of the signature and nomogram. Both c-index and AUC of nomogram exceeded the immune-related signature, especially in predicting 5-year OS (Tables 4 and 5), which indicated the nomogram had better predictive power in long-term follow-up. Therefore, through combination of the 8-lncRNA signature and other prognosis-related clinical factors, the nomogram further increased the predictive power of survival.

**Table 5:** Area under curve (AUC) from time-dependent receiver operating characteristic (ROC) curves.

	**AUC (95%CI)**		**Z-test**	***P*-value**
	**8-lncRNA Risk score**	**Nomogram**		
**Training cohort**				
1-Year	0.764 (0.721-0.807)	0.746 (0.700-0.792)	-0.564	0.573
3-Year	**0.756 (0.720-0.792)**	**0.829 (0.793-0.865)**	**2.798**	**0.005**
5-Year	**0.742 (0.704-0.780)**	**0.825 (0.781-0.869)**	**2.777**	**0.006**
**Testing cohort**				
1-Year	**0.797 (0.748-0.846)**	**0.660 (0.581-0.739)**	**-2.900**	**0.004**
3-Year	0.739 (0.678-0.799)	0.673 (0.611-0.735)	-1.491	0.136
5-Year	**0.724 (0.651-0.797)**	**0.801 (0.759-0.843)**	**2.302**	**0.021**
**Entire TCGA BLCA cohort**				
1-Year	0.742 (0.710-0.774)	0.778 (0.748-0.808)	1.608	0.108
3-Year	0.769 (0.732-0.806)	0.755 (0.720-0.790)	-0.537	0.591
5-Year	0.730 (0.685-0.775)	0.750 (0.704-0.796)	0.612	0.541

In recent years, immunotherapy for BCa attracted more and more attention owing to the availability of targeted immunotherapies and checkpoint inhibitors [33–35]. Among these eight lncRNAs, WNT5A-AS1 and AL136084.3 are risk lncRNAs, while MIF-AS1, AC008735.2, AL357033.4, LINC00649, AC099343.2 and USP30-AS1 are protective lncRNAs. MIF-AS1 acted as a competing endogenous RNA by activating miR-1249-3p/HOXB8 axis in breast cancer [36]. LINC00649 might participate in intracellular receptor signaling pathways in prostate cancer patients [37]. LINC00649 was reported to be involved in vacuolar transport and histone modification functions as well as G protein-coupled receptor and Rho GTPases signaling pathways and is significantly related to the development and prognosis of CRC [38]. Although most of the identified lncRNAs have not been reported to be directly associated with immune-related function, the eight lncRNAs might participate in immune system through indirect pathways for the reason that enrichment results were immune-related. Hence, the eight lncRNAs targeting immune-related genes will have attractive applications to provide therapeutic targets for BCa, which could improve the prognosis of BCa patients.

Nowadays, many studies used high-throughput sequencing data to identify gene signatures and construct clinical predictive models. Gao et al. [39] identified a 6-lncRNA signature to predict the prognosis of BCa and their AUC for 5-year survival is 0.751, which is similar to 0.742 by our signature. However, they didn’t report the AUC value for 1-year survival and thus the predictive power for short-term follow-up is uncertain. In addition, we validated its application in the external validation cohort, which further confirmed the predictive value of our signature. We also constructed a nomogram by enrolling the immune-related signature and other prognosis-related clinical factors to broaden the applicability and increase the clinical significance. In the present study, we validated our findings in other online databases and discussed the relationship between immune system and BCa by immunohistochemistry.

To our knowledge, our study focused on lncRNAs targeting immune-related genes and further construct a signature and a nomogram for BCa. However, several limitations still existed. Firstly, most enrolled patients from TCGA BLCA dataset are MIBC and only 2 patients are NMIBC (stage I). Hence, the 8-lncRNA signature and nomogram might be more suitable for MIBC patients and further cohorts based on NMIBC patients are required to verify its application in NMIBC management in the future. Secondly, although external validation has been performed to confirm the predictive power of the signature and nomogram, the exact molecular mechanisms of eight lncRNAs have not been investigated in the present study. Therefore, further in vitro and in vivo studies based on functional studies are warranted to verify these hypotheses and to make these results more convincible for clinical application in the future.

In summary, we identified an immune-related signature and nomogram based on eight lncRNAs targeting immune-related genes in MIBC. The 8-lncRNA signature and nomogram were confirmed to be independent prognostic indicators for MIBC and could act as a good classifier in different subgroups of MIBC patients. External validation was utilized to verify the predictive value and immunohistochemistry as well as GSEA validated the association between the signature and immune-related functions. These eight lncRNAs will have attractive applications to provide new diagnostic methods and treatment targets for MIBC, which could improve the prognosis of MIBC patients, if validated by further experiments.

## MATERIALS AND METHODS

### TCGA BLCA dataset and functional enrichment analysis

TCGA BLCA dataset contained BCa samples (n = 411) and normal bladder samples (n = 19). The RNA-sequencing data and clinical data were downloaded from TCGA (http://tcga-data.nci.nih.gov/tcga/) database.

To identify DEGs between BCa samples and normal bladder samples in TCGA BLCA dataset, we utilized limma R package [40]. The cut-off criteria of adjusted *P*-value (*adj. P-*value) was set as 0.05 and the criterion of Fold change was set as |logFC| ≥ 1. We also generated a volcano plot for these DEGs.

In order to reveal the potential functions of DEGs, the clusterProfiler R package is used to perform GO enrichment analysis and KEGG pathway enrichment analysis. In addition, *adj. P-*value < 0.05 was set as the cutoff criteria.

### Immune-related genes and functional enrichment analysis

Immune-related genes were extracted from Molecular Signatures Database v4.0 (http://www.broadinstitute.org/gsea/msigdb/index.jsp: Immune system process M13664, Immune response M19817) [41]. Ultimately, 332 immune-related genes were identified. Then, we identified immune-related DEGs between BCa samples and normal bladder samples in TCGA BLCA dataset and generated a volcano plot as well as a heat map for these immune-related DEGs by using pheatmap R package. In addition, GO and KEGG pathway enrichment analyses were carried out with the criteria mentioned above.

Venn diagrams (http://bioinformatics.psb.ugent.be/webtools/Venn/) were applied to identify overlapped enrichment results between all DEGs enrichment analysis and immune-related DEGs enrichment analysis. KEGG pathway and GO functional enrichments including BP, cell component (CC) and molecular function (MF) were calculated respectively.

### Identification of lncRNAs targeting immune-related genes

A total of 33 immune-related DEGs (18 were up-regulated and 15 were down-regulated) were identified through differential expression analysis based on the 332 immune-related genes. In order to further find lncRNAs targeting immune-related genes, Pearson correlation analysis was performed between expression levels of all lncRNAs and 18 up-regulated immune-related DEGs or 15 down-regulated immune-related DEGs, respectively. |R| > 0.5 and *P-*value < 0.01 were established as cut-off criteria. Ultimately, 262 lncRNAs targeting immune-related genes were identified according to the above criteria in the TCGA BLCA dataset.

### Identification of an immune-related prognostic signature based on lncRNAs targeting immune-related genes

We used survival R package to construct the lncRNA signature. The criteria for BCa samples in identification and validation of the lncRNA signature were as follows: (1) complete lncRNA expression values and clinical characteristics (age at diagnosis, gender, UICC stage, histological grade, and survival time); and (2) samples with total survival time less than 1 month were excluded for these samples might die of nonneoplastic factors including severe infection or hemorrhage. Ultimately, a total of 389 BCa samples were included for further construction of the lncRNA signature. We randomly divided 389 BCa samples into the training cohort (n = 195) and testing cohort (n = 194). The training cohort was used to develop the lncRNA signature. The testing cohort was used to validate the lncRNA signature.

Next, the 262 selected lncRNAs were put into univariate Cox regression analysis in the training cohort in order to screen prognosis-related lncRNAs (*P-*value < 0.01). Univariate Cox regression analysis identified 21 prognosis-related lncRNAs. Then, we used stepwise selection with Akaike information criteria (AIC) method from the 21 prognosis-related lncRNAs to select the optimal model [42]. Ultimately, we selected 8 lncRNAs with the smallest AIC value to construct the predictive model.

We performed a multivariate Cox regression analysis on the 8 prognosis-related lncRNAs to develop a prognostic lncRNA signature and calculate the coefficients [43–45]. The risk score of the prognostic lncRNA signature for each patient was calculated as the following formula:

Risk score = Expression_lncRNA1_ × Coefficient_lncRNA1_ + Expression_lncRNA2_ × Coefficient_lncRNA2_ + … + Expression_lncRNAn_ × Coefficient_lncRNAn_.

The risk score of the prognostic lncRNA signature was calculated according to a linear combination of the expression level of lncRNAs weighted by the regression coefficient (β). The β was calculated by log-transformed HR derived from multivariate Cox regression analysis [46, 47]. Low-risk and high-risk groups were determined by the median value of risk score.

### Predictive power of the lncRNA prognostic signature in training cohort and validation

We performed univariate and multivariate Cox regression analysis to evaluate the predictive power of lncRNA signature and other clinical characteristics (age, gender, UICC stage, and histological grade) in training cohort (n = 195). C-index and time-dependent ROC curve with AUC value were performed to further assess the predictive value of lncRNA signature and these clinical characteristics by survivalROC, timeROC and rms R packages. The training cohort was divided into two low-risk and high-risk groups by the median value of risk score. Then, KM survival curve was plotted to compare the differences of OS in the two groups. *P*-value < 0.05 was considered as statistically significant difference.

At the same time, stability and reliability of the lncRNA signature was validated in testing cohort (n = 194) and in entire TCGA BLCA cohort (n = 389). They were also divided into two low-risk and high-risk groups by the median value of risk score. Furthermore, univariate and multivariate Cox regression analysis, time-dependent ROC curve, KM survival curve and c-index were analyzed for the validation as mentioned earlier.

### Bioinformatics analysis

PCA was carried out to profile expression patterns of grouped samples by using scatterplot3d R package. GSEA (http://www.broadinstitute.org/gsea/index.jsp) was carried out to find different functional phenotypes between the two groups [48]. Gene set permutations were performed 1000 times for each analysis. FDR < 25% and nominal *P*-value < 0.05 were regarded as the cut-off criteria of sorting GO and KEGG pathway enrichments in GSEA.

### Immunohistochemistry and survival analysis of immune-related DEGs

Immunohistochemistry was obtained from THPA (http://www.proteinatlas.org/) [49]. We evaluated expression levels of 4 immune-related DEGs including CTSG (Cathepsin G), CXCL12 (chemokine [C-X-C motif] ligand 12), LIG1 (DNA Ligase 1) and TBX1 (T-Box Transcription Factor 1) between normal bladder tissues and BCa tissues from THPA.

What’s more, we used GEPIA database (http://gepia.cancer-pku.cn/) for further validating the differential expression of the 4 immune-related genes based on TCGA and GTEx datasets and calculating OS with the 4 immune-related genes on the basis of TCGA BLCA dataset [50].

### Identification and validation of a predictive nomogram

Nomogram [51] was constructed by including age, UICC stage, histological grade, and the immune-related prognostic signature in training cohort. The training cohort was divided into two low-nomogram-score and high-nomogram-score groups by the median value of nomogram score. KM survival curve, time-independent ROC curve and c-index were conducted to evaluate the predictive power of nomogram. We also performed calibration plot to explore the calibration and the discrimination of the nomogram by a bootstrap method with 1000 resamples.

In the meantime, stability and reliability of the nomogram was validated in testing cohort. KM survival curve, ROC curve and c-index were analyzed for the validation in testing cohort (n = 194) and in entire TCGA BLCA cohort (n = 389) as mentioned earlier.

### Quantitative real-time polymerase chain reaction and External validation in Tianjin cohort

A total of 72 patients who were pathologically and clinically diagnosed with MIBC were enrolled from Tianjin Medical University General Hospital. MIBC tissues were obtained at the time of first surgery as Tianjin cohort. The study was approved by the Ethics Committee of Tianjin Medical University General Hospital. All recruited participants signed informed consent before being enrolled in our study.

Total RNA from MIBC samples were extracted using RNeasy kit (Qiagen, Valencia, CA). The first chain of cDNA was synthesized by reverse transcription with TaqMan^®^ Reverse Transcription Reagents (Applied Biosystems, Grand Island, NY). GAPDH was used as internal control. The sequences of the primers were displayed in Table 7. qRT-PCR was performed using the CFX96 Touch PCR system (Bio-Rad). The relative lncRNA expression levels of WNT5A-AS1, AL136084.3, MIF-AS1, AC008735.2, AL357033.4, LINC00649, AC099343.2 and USP30-AS1 were calculated by 2^-ΔΔCt^ method. Risk score was calculated with the previous following formula of signature. In addition, univariate and multivariate Cox regression analysis, time-dependent ROC curve and KM survival curve were analyzed for the validation in Tianjin cohort (n = 72) as mentioned earlier.

**Table 7:** Primer sequences used to amplify target lncRNAs by quantitative real-time polymerase chain reaction (qRT-PCR).

**lncRNA name**		**Primer sequences**
WNT5A-AS1	Forward	5'-AAAACGCACAAGTCGCCATC-3'
	Reverse	5'-CCGCACAGCAATAAGTTCCG-3'
AL136084.3	Forward	5'-GCTGCCTTATGTAACCTGCG-3'
	Reverse	5'-AAGAGTGCTTTCTTGCGGGT-3'
MIF-AS1	Forward	5'-CACTGTGGTCCCGCCTTTTG-3'
	Reverse	5'-CTAGCCGCCAAGTGGAGAAC-3'
AC008735.2	Forward	5'-CAAATATGAAACTGCCACAGAGAGG-3'
	Reverse	5'-TTACTATTGACTTCTACACCCCCAC-3'
AL357033.4	Forward	5'-AATGATGTCTGGTCCGCGTT-3'
	Reverse	5'-CTGCAATGTCCTGTTCCCCT-3'
LINC00649	Forward	5'-GTTATTGTCAACGCCAGCCC-3'
	Reverse	5'-GGTTGTCTCGGACCTCATGG-3'
AC099343.2	Forward	5'-TAGACCAGGCGGTGGATAGT-3'
	Reverse	5'-GAATCCTGAATCTGCGTGCG-3'
USP30-AS1	Forward	5'-ATACGACGGTTCCCGAGACA-3'
	Reverse	5'-GACGTGGTCCGTCAGCTATT-3'
GAPDH	Forward	5'-AGAAGGCTGGGGCTCATTTG-3'
	Reverse	5'-AGGGGCCATCCACAGTCTTC-3'

### Statistical analysis

Statistical analyses were carried out using R software (v3.5.3: http://www.r-project.org) and SPSS v23.0. The RNA-sequencing data of gene and lncRNA were log2-transformed. Univariate and multivariate Cox regression analyses, KM method and log-rank test were used to compare the influence of the lncRNA prognostic signature on survival along with other clinical characteristics. Chi-square test and Student’s t test were used to evaluate qualitative variables and quantitative variables, respectively. Time-dependent ROC curve and c-index was utilized to assess the prognostic value based on the lncRNA signature and nomogram. Delong’s Z-test was utilized to compare the AUC and c-index between the signature and nomogram [52]. The two-sided *P*-value < 0.05 was regarded as statistically significant.
